# Optimization of reaction parameters in hydrothermal synthesis: a strategy towards the formation of CuS hexagonal plates

**DOI:** 10.1186/1752-153X-7-67

**Published:** 2013-04-10

**Authors:** Yow Loo Auyoong, Pei Lay Yap, Xing Huang, Sharifah Bee Abd Hamid

**Affiliations:** 1 COMBICAT Laboratory, Nanotechnology & Catalysis Research Centre (NANOCAT), University of Malaya, Kuala Lumpur 50603, Malaysia; 2 Chemistry Department, Faculty of Science, University of Malaya, Kuala Lumpur 50603, Malaysia; 3 Key Laboratory of Photochemical Conversion and Optoelectronic Materials, Technical Institute of Physics and Chemistry, Chinese Academy of Sciences, Beijing 100190, China

## Abstract

**Background:**

For decades, copper sulphide has been renowned as the superior optical and semiconductor materials. Its potential applications can be ranged from solar cells, lithium-ion batteries, sensors, and catalyst systems. The synthesis methodologies of copper sulphide with different controlled morphology have been widely explored in the literature. Nevertheless, the understanding on the formation chemistry of CuS is still limited. The ultimate approach undertaking in this article is to investigate the formation of CuS hexagonal plates via the optimization of reaction parameters in hydrothermal reaction between copper (II) nitrate and sodium thiosulphate without appending any assistant agent.

**Results:**

Covellite (CuS) hexagonal plates were formed at copper ion: thiosulphate ion (${\mathrm{Cu}}^{2+}:{\text{S}}_{2}{\text{O}}_{3}{}^{2-}$) mole ratio of 1:2 under hydrothermal treatment of 155°C for 12 hours. For synthesis conducted at reaction temperature lower than 155°C, copper sulphate (CuSO_4_), krohnite (NaCu_2_(SO_4_)(H_2_O)_2_] and cyclooctasulphur (S_8_) were present as main impurities with covellite (CuS). When ${\mathrm{Cu}}^{2+}:{\text{S}}_{2}{\text{O}}_{3}{}^{2-}$ mole ratio was varied to 1: 1 and 1: 1.5, phase pure plate-like natrochalcite [NaCu_2_(SO_4_)(H_2_O)] and digenite (Cu_9_S_5_) were produced respectively. Meanwhile, mixed phases of covellite (CuS) and cyclooctasulphur (S_8_) were both identified when ${\mathrm{Cu}}^{2+}:{\text{S}}_{2}{\text{O}}_{3}{}^{2-}$ mole ratio was varied to 1: 2.5, 1: 3 and 1: 5 as well as when reaction time was shortened to 1 hour.

**Conclusions:**

CuS hexagonal plates with a mean edge length of 1 μm, thickness of 100 nm and average crystallite size of approximately (45 ± 2) nm (Scherrer estimation) were successfully synthesized via assisting agent- free hydrothermal method. Under a suitable ${\mathrm{Cu}}^{2+}:{\text{S}}_{2}{\text{O}}_{3}{}^{2-}$ mole ratio, we evidenced that the formation of covellite (CuS) is feasible regardless of the reaction temperature applied. However, a series of impurities were attested with CuS if reaction temperature was not elevated high enough for the additional crystallite phase decomposition. It was also identified that ${\mathrm{Cu}}^{2+}:{\text{S}}_{2}{\text{O}}_{3}{}^{2-}$ mole ratio plays a vital role in controlling the amount of cyclooctasulphur (S_8_) in the final powder obtained. Finally, reaction time was recognized as an important parameter in impurity decomposition as well as increasing the crystallite size and crystallinity of the CuS hexagonal plates formed.

## Background

Copper sulphides have received momentous attention from both chemists and material scientists owing to its unique physical and chemical properties [1-3] for potential applications in solar cells, lithium-ion batteries, sensors, and catalysts [4-8]. To date, various copper sulphide of specific morphologies such as flakes-like [9], rod-like [10], needle-like [11], wires-like [12,13], tubes-like [14], or even spheres-like [15] have been reported. Nonetheless, the significance of plate-like structured materials is remarkably manifested in the literature as promising building blocks for nanodevices with its controlled crystal orientation due to their anisotropic structures [16-19]. In fact, a great deal of effort has been dedicated to the synthesis of CuS with plate-like structure, particularly hexagonal plate-shaped. Du et al. have revealed that shape-controlled hexagonal CuS can be prepared by employing copper acetate and carbon disulphide with toluene and hexadecylamine as assisting agents via a solvothermal process [20]. CuS hexagonal plates were fabricated using CTAB and nitric acid as assisting agent on top of copper (II) chloride and sodium thiosulphate as precursors via a hydrothermal method [21]. Y. Liu et al. have synthesized CuS hexagonal plates by applying hexadecylamine, ethanol, potassium ethylxanthate and copper nitrate through a facile solution route [22]. A mixed-mode method of wet chemical and modified hydrothermal techniques which employed copper (II) chloride, acetylacetone, sodium acetate, dichloromethane, ethanol, and sodium hydroxide as precursors was also demonstrated by Basu et al. to achieve the formation of CuS with hexagonal stacked plates morphology [23]. Despite the enormous synthesis methods developed in fabricating hexagonal shaped CuS, it could be seen that the formation of CuS is not fully understood due to the use of multiple solvent phases or additional assisting agent which is present in the reactions. This factor can eventually result in a complicated series of reaction occurring in the synthesis which leads to difficulty in explaining the CuS formation.

Generally, the ultimate challenge in any synthesis approach is to identify the role of each reaction parameter in controlling the morphology and crystal structure of the final products obtained. This understanding is essential in distinguishing and establishing the reaction mechanism for the targeted compound formation. Hence, our approach is focusing on the investigation of CuS hexagonal plates formation via the optimization of reaction parameters in hydrothermal reactions between copper (II) nitrate and sodium thiosulphate without appending any assistant agent. Hydrothermal method is selected in this work because it requires no complex organometallic precursor in the reaction [24,25]. Furthermore, highly crystalline products with controlled morphology can be easily achieved in hydrothemal treatment by varying the specific source species, reaction temperature, reaction time and etc. [26,27]. In the entire methodology, we have chosen mild reactants like copper (II) nitrate and sodium thiosulphate as the precursors; unlike the case of hydrogen sulphide [28,29], ammonium sulphide [30] and sodium sulphide [14,31,32] which are highly reactive and nasty in handling during the experiment.

In the present study, covellite (CuS) is identified as a thermodynamically stable compound in which its formation is feasible at appropriate ${\mathrm{Cu}}^{2+}:{\text{S}}_{2}{\text{O}}_{3}{}^{2-}$ mole ratio even at room temperature condition. However, a series of impurities were attested with CuS if reaction temperature was not elevated high enough for their decompositions. With the aim to further comprehend CuS hexagonal plates formation, the roles of reaction temperature, ${\mathrm{Cu}}^{2+}:{\text{S}}_{2}{\text{O}}_{3}{}^{2-}$ mole ratio and reaction time towards the crystal structures and morphologies of the final products formed were systematically investigated. The possible formation and growth of CuS hexagonal plates during the hydrothermal treatment were also proposed based on the presence of Cu (I) instead of Cu (II) species in the crystal structure of covellite (CuS) [33]. The entire methodology described herein has provided us further insight on the use of facile hydrothermal technique in studying the reactions between aqueous solution phase reactants as well as synthesizing highly crystalline phase pure covellite (CuS) hexagonal plates without any assisting agent.

## Results and discussion

### Structural and compositional analyses of CuS hexagonal plates

**Figure 1:**
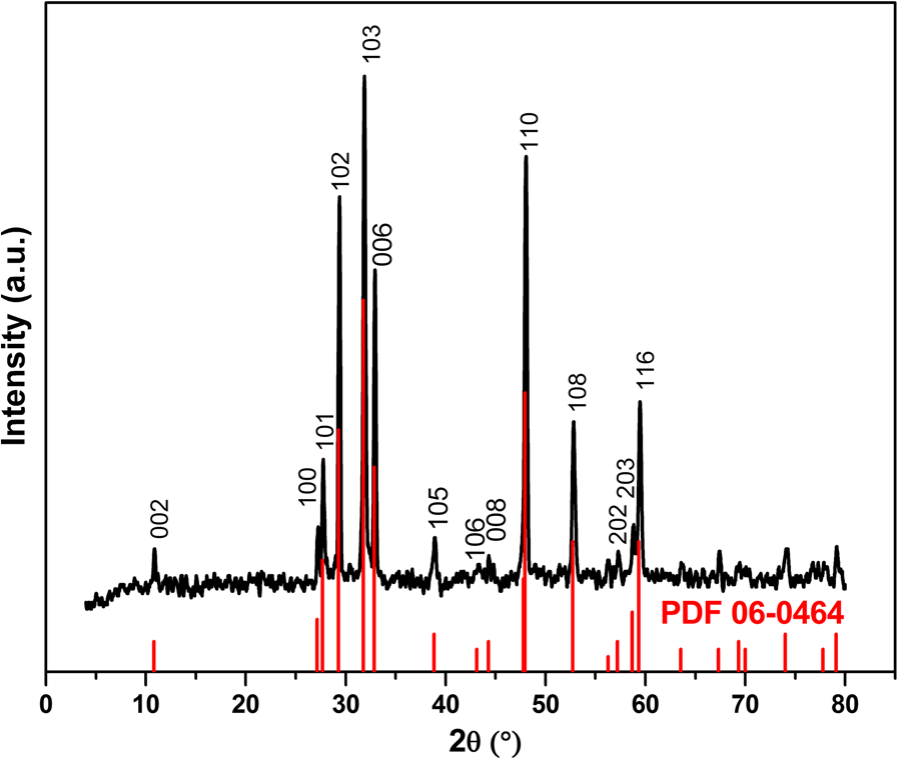
**Powder XRD pattern of CuS.** Synthesis was conducted at ${\mathrm{Cu}}^{2+}:{\text{S}}_{2}{\text{O}}_{3}{}^{2-}$ mole ratio of 1: 2 at 155°C for 12 hours.

The crystal phase of the product formed in the reaction of copper nitrate and sodium thiosulphate was identified by powder XRD technique. The powder XRD pattern as illustrated in Figure 1 shows the crystal phase of product formed at ${\mathrm{Cu}}^{2+}:{\text{S}}_{2}{\text{O}}_{3}{}^{2-}$ mole ratio of 1: 2 under 155°C. All the characteristic peaks in this pattern correspond well to the hexagonal phase covellite in the space group of *P63/mmc* which can be well indexed to PDF 06–464 with a = 3.792 Å and c = 16.34 Å. The diffractogram of this compound exhibits no XRD peaks arising from impurities of CuO, S, and other Cu_x_S. This denotes high phase purity of the CuS obtained in the facile hydrothermal batch route developed.

**Figure 2:**
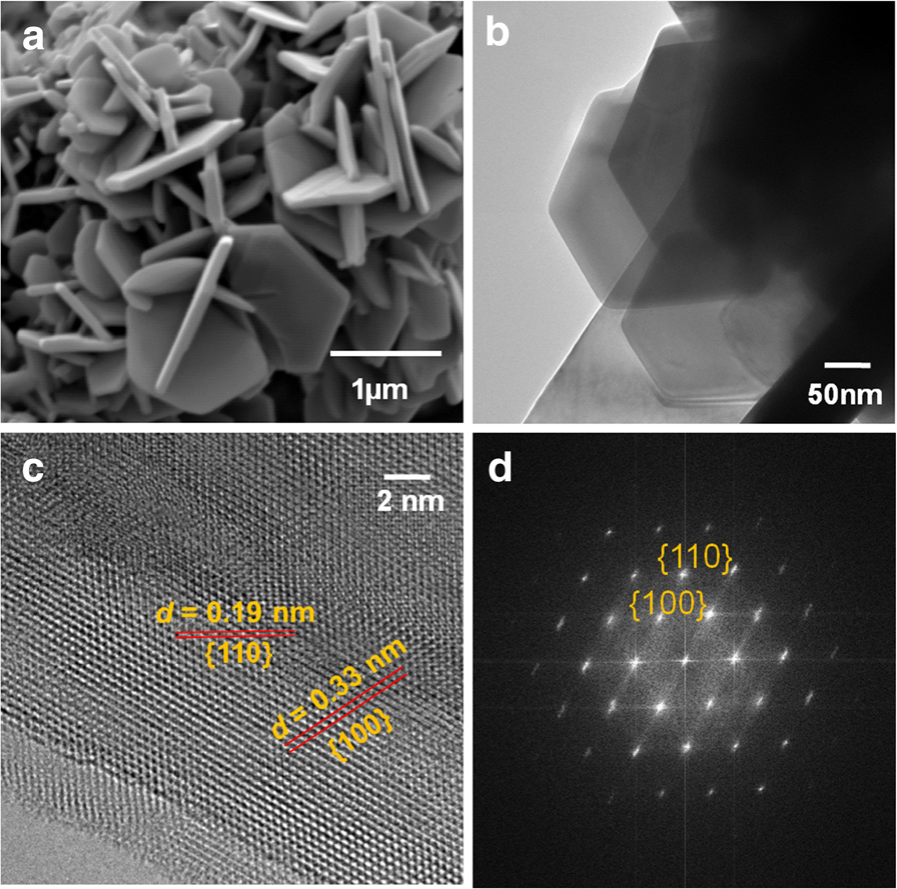
**Electron Micrographs of CuS hexagonal plates.** FESEM image (**a**), TEM image (**b**), HRTEM image with incident beam from the <001 > direction (**c**) and Fast Fourier Transform (FFT) pattern (**d**) of CuS hexagonal plates.

The morphology of the fabricated sample was characterized by FESEM technique (Figure 2a). It is clearly shown that the powder obtained exhibits particles with hexagonal plate structure. The hexagonal shape plates assembled, interlaced and perpendicular to one another. It could be observed that the hexagonal plates have a mean edge length of 1 μm and an average thickness of *ca.* 100 nm. The hexagonal plate architecture found can be significantly related to the formation of the hexagonal phase of covellite in which it correlates well with the single phase of covellite determined by powder XRD analysis. The insight of the hexagonal plate microstructure was also examined by employing TEM and HRTEM analyses. The TEM image as depicted in Figure 2b vividly shows the stack layers orientation of many CuS plates with hexagonal structure. The observation of hexagonal shaped particle from TEM analysis agrees well with the morphology determined from FESEM images. Figure 2c depicts the HRTEM image of the hexagonal shaped particle. From the well resolved 2D lattice fringes of the CuS hexagonal plate measured, two adjacent lattice spacings of 0.19 nm and 0.33 nm have been identified from the image. It is important to note that both of the lattice spacings of 0.19 nm and 0.33 nm relate well to the {110} and {100} plane spacings of hexagonal CuS respectively. A Fast Fourier Transform (FFT) pattern of the as-synthesized CuS hexagonal plate is also captured in Figure 2d. The ordered hexagonal-like spot arrays visibly illustrated in the FFT pattern again confirmed the formation of CuS with hexagonal shape. All these results strongly signify the single crystallinity of the CuS hexagonal plates formed in this hydrothermal synthesis.

**Figure 3:**
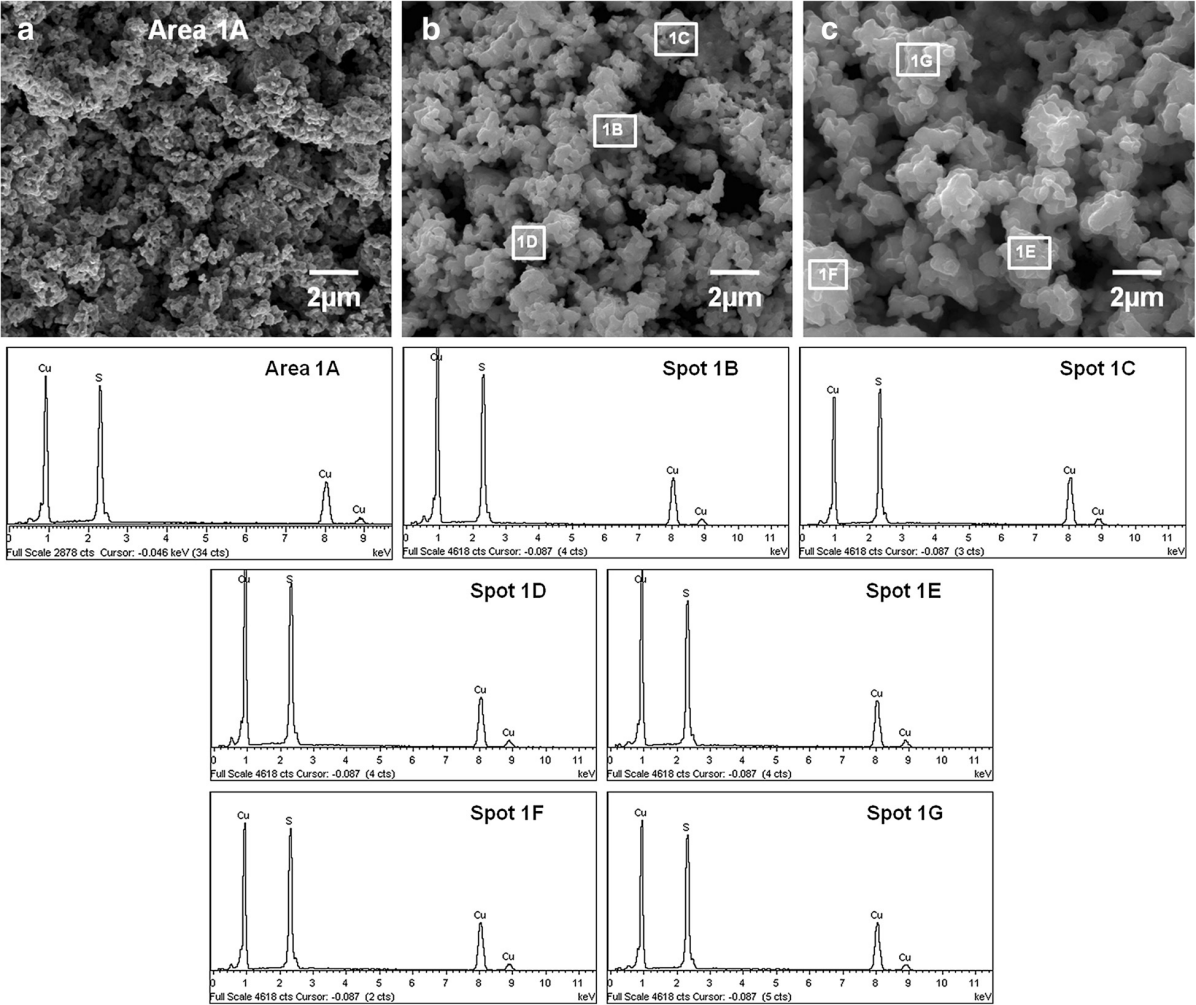
**EDX analysis on CuS hexagonal plates.** Area analysis (**a**), spot analysis (**b**) and (**c**) of CuS hexagonal plates. The related EDX spectra are labelled as Area 1A, Spot 1B to Spot 1G.

The bulk elemental composition of the as-synthesized powder was analyzed by X-ray fluorescence (XRF) technique. XRF analysis revealed that the bulk composition of the respective powder consists of only Cu and S in which the Cu: S atomic ratio calculated is 1: 1.04. In addition, EDX analysis was conducted to probe the localized surface elemental composition of the hexagonal plates. Figure 3 depicts the multiple EDX analysis scanned at one area and different spots for the hexagonal plates synthesized. From the numerous scans of EDX spectra, the powder is found to compose Cu and S elements in which no significant contamination from other elements can be detected. As tabulated in Table 1, the respective quantified EDX spectra disclose the average atomic composition of Cu: S is closed to each other, reaching average percentage of approximately 50: 50. Both of the results attained from XRF and EDX analyses are complementary to each other and the atomic ratio of Cu and S evaluated is consistent with the ideal nominal stoichiometric ratio of covellite which is 1: 1 for Cu: S. Therefore, the bulk and localized distributions of Cu and S are in good agreement with the powder XRD and HRTEM analysis in which merely pure phase covellite (CuS) is found in the hexagonal plates.

**Table 1:** EDX analysis on CuS hexagonal plates

**Area/ Spot**	**Cu Atomic %**	**S Atomic %**
Area 1A	51.53	48.47
Spot 1B	48.28	51.72
Spot 1C	51.40	48.60
Spot 1D	48.08	51.92
Spot 1E	49.11	50.89
Spot 1 F	50.55	49.45
Spot 1G	50.24	49.76
**Average**	**49.88**	**50.12**

Synthesis was conducted at 155°C with ${\mathrm{Cu}}^{2+}:{\text{S}}_{2}{\text{O}}_{3}{}^{2-}$ mole ratio of 1: 2 for 12 hours with reference to Figure 3.

### Role of reaction temperature in hydrothermal synthesis

**Figure 4:**
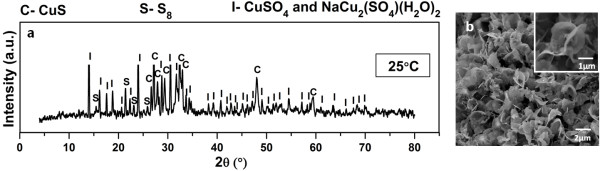
**Product prepared at 25°C.** Powder XRD pattern (**a**) and FESEM images (**b**) of product formed at 25°C with ${\mathrm{Cu}}^{2+}:{\text{S}}_{2}{\text{O}}_{3}{}^{2-}$ mole ratio of 1: 2 for 12 hours.

**Figure 5:**
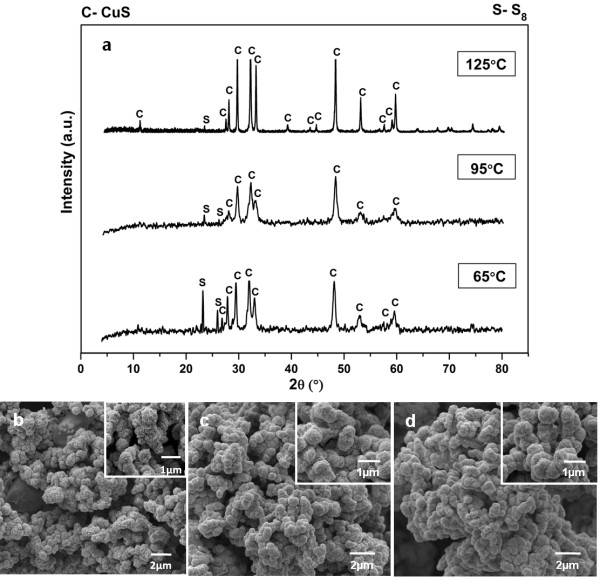
**Products prepared at different reaction temperatures.** Powder XRD patterns (**a**), FESEM images of products synthesized with ${\mathrm{Cu}}^{2+}:{\text{S}}_{2}{\text{O}}_{3}{}^{2-}$ mole ratio of 1: 2 for 12 hours at 65°C (**b**), 95°C (**c**) and 125°C (**d**).

**Figure 6:**
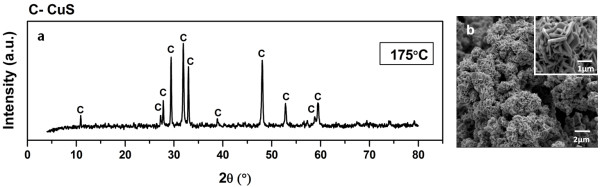
**Product prepared at 175°C.** Powder XRD pattern (**a**) and FESEM images (**b**) of product synthesized at 175°C with ${\mathrm{Cu}}^{2+}:{\text{S}}_{2}{\text{O}}_{3}{}^{2-}$ mole ratio of 1: 2 for 12 hours.

In order to attain a better understanding on the formation of phase pure CuS hexagonal plate, several experiments were carried out at different synthesis temperatures with ${\mathrm{Cu}}^{2+}:{\text{S}}_{2}{\text{O}}_{3}{}^{2-}$ mole ratio fixed at 1: 2 for 12 hours. Figures 4, 5 and 6 depict the powder XRD patterns of crystalline compounds formed at 25.0, 65.0, 95.0, 125.0 and 175.0°C. At reaction temperature of 25°C, mixed phases of copper sulphate [CuSO_4_], krohnite [NaCu_2_(SO_4_)(H_2_O)_2_], cyclooctasulphur [S_8_] as well as covellite [CuS] are observed in the powder XRD diffractogram. This observation strongly suggests that CuS is a thermodynamically stable compound in which under a suitable ${\mathrm{Cu}}^{2+}:{\text{S}}_{2}{\text{O}}_{3}{}^{2-}$ mole ratio (specifically 1: 2 in this study), its formation is feasible even under room temperature condition. The morphology of this mixed phase powder is shown in Figure 4b. The morphology of this sample resembles the shape of paper flower with many uniform clusters. Each cluster of the flower is surrounded by 3 or 4 thin bracts of petals.

As reaction temperature was increased to 65.0°C, both of the CuS and S_8_ phases are identified while NaCu_2_(SO_4_)(H_2_O)_2_ and CuSO_4_ disappeared from the diffractogram. For NaCu_2_(SO_4_)(H_2_O)_2_ and CuSO_4_ phases, they are determined to be slightly and highly soluble in water respectively [34,35]. For both CuS (K_sp_ = 8 x 10^-37^) and S_8_, they tend to have low solubility in water even with solution temperature elevation [36,37]. A preferential dissolution of NaCu_2_(SO_4_)(H_2_O)_2_ and CuSO_4_ occurred in water during the reaction. Thus, the retention of both CuS and S_8_ as well as the disappearance of NaCu_2_(SO_4_)(H_2_O)_2_ and CuSO_4_ in the product are closely related to their solubility in water at increasing temperature. The morphology of the sample has changed from the flower-like (at 25.0°C) to ball-like structures as indicated in Figure 5b. As reaction temperature was further increased to 95.0 and 125.0°C, the CuS phase is still accompanied by S_8_ phase in the powder XRD patterns. Even with relatively higher temperature applied, the crystalline S_8_ peak at 2θ = 23° is becoming less intense as compared to the diffractogram at 65.0°C. This indicates that S_8_ impurity has not been completely decomposed even at 125°C. Moreover, the morphology of the ball-like structures synthesized at 95.0 and 125.0°C remained similar as the product formed at 65.0°C. This result signifies that the growth of hexagonal plate-like structure cannot be achieved if reaction temperature is not elevated high enough to decompose the S_8_ impurity phase.

**Figure 7:**
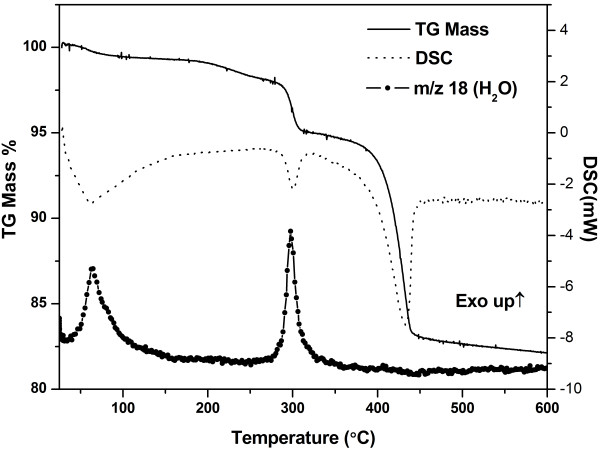
**Thermal decomposition of CuS hexagonal plates in the absence of oxidative condition.** TG-MS-DSC curves on CuS hexagonal plates under 100% Ar gas.

In fact, the decomposition of S_8_ is estimated at 149.5°C in which a complete breaking of crown S_8_ ring might be expected [38]. In order to investigate the optimum temperature in eliminating S_8_ phase without affecting CuS phase in the powder, phase pure CuS hexagonal plates obtained at ${\mathrm{Cu}}^{2+}:{\text{S}}_{2}{\text{O}}_{3}{}^{2-}$ mole ratio of 1: 2 was subjected to thermal treatment under a controlled flow of 100% Ar gas. From the TG-DSC curves (Figure 7), the thermal decomposition of CuS can be divided into three major steps based on the DSC endotherm observed. The first decomposition step is identified in the temperature range of 38.0 – 165.0°C in which a shallow broad DSC endotherm is detected. This event is attributed to desorption of physisorbed water from CuS crystal as the mass-to-charge ratio (m/z) of 18 was evidenced from the MS signal. The second decomposition step is found in the temperature range of 217.0 – 348.0°C in which a well-defined DSC endotherm is associated. This event can be related well to desorption of crystallization water as mass-to-charge ratio (m/z) of 18 was again evidenced from the MS signal. The third decomposition step is identified in the temperature range of 345.0 – 470.0°C in which a huge mass loss of the sample (≈ 12.0%) as well as the sharp DSC endotherm is observed. Since no significant MS signal is detected, an endothermic event that involves the crystallographic changes from CuS phase to other Cu_x_S phases is deduced. From the analysis, it is obvious that the as-formed CuS phase at 25.0°C will not be affected if reaction temperature is maintained below 300.0°C. Furthermore, the synthesis conducted at 175°C also discloses that the microstructure of CuS hexagonal plates (Figure 6) remains as no obvious change in the morphology can be detected at this temperature. Thus, these results strongly suggest that the CuS hexagonal phase is retained as a single phase product if the decomposition of S_8_ ring impurity is performed in the range of 155.0 – 300.0°C. Therefore, the highlighted observations have shown that reaction temperature plays an important role in controlling the phase purity, crystal phase as well as morphology of the final product in the hydrothermal synthesis developed.

### Role of ${\text{Cu}}^{2+}:{\text{S}}_{2}{\text{O}}_{3}{}^{2-}$ mole ratio in hydrothermal synthesis

**Figure 8:**
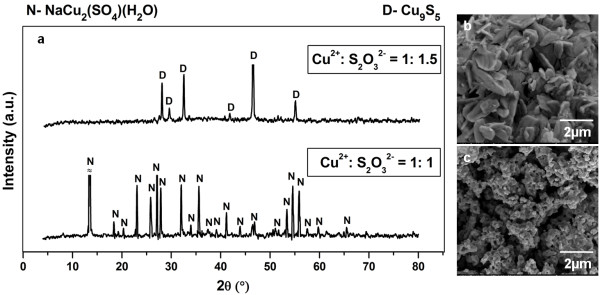
**Products prepared at different **${\mathrm{Cu}}^{2+}:{\text{S}}_{2}{\text{O}}_{3}{}^{2-}$**mole ratio.** Powder XRD patterns (**a**), FESEM images of products synthesized at 155°C for 12 hours with ${\mathrm{Cu}}^{2+}:{\text{S}}_{2}{\text{O}}_{3}{}^{2-}$ mole ratio of 1: 1 (**b**) and 1: 1.5 (**c**).

**Figure 9:**
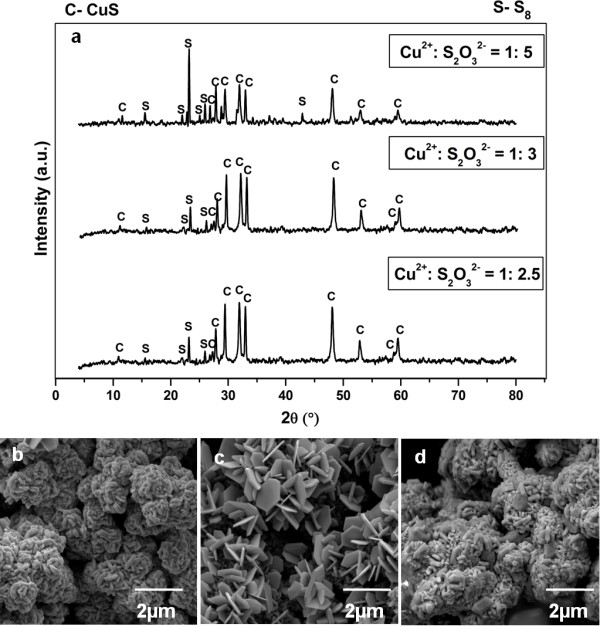
**Products prepared at different **${\mathrm{Cu}}^{2+}:{\text{S}}_{2}{\text{O}}_{3}{}^{2-}$**mole ratio.** Powder XRD patterns (**a**), FESEM images of products synthesized at 155°C for 12 hours with ${\mathrm{Cu}}^{2+}:{\text{S}}_{2}{\text{O}}_{3}{}^{2-}$ mole ratio of 1: 2.5 (**b**), 1: 3 (**c**) and 1: 5 (**d**).

It has been identified previously that the variation of synthesis temperature is vital in directing the final crystal phase and morphology of the product. It is also found that pure CuS hexagonal phase can only be achieved if the synthesis is performed at ${\mathrm{Cu}}^{2+}:{\text{S}}_{2}{\text{O}}_{3}{}^{2-}$ mole ratio of 1: 2 under the reaction temperature of 155.0°C. In this section, we further investigated the role of ${\mathrm{Cu}}^{2+}:{\text{S}}_{2}{\text{O}}_{3}{}^{2-}$ mole ratio in the formation of copper sulphide via the variation of ${\mathrm{Cu}}^{2+}:{\text{S}}_{2}{\text{O}}_{3}{}^{2-}$ mole ratio under the typical synthesis condition. The powder XRD patterns of the products formed at ${\mathrm{Cu}}^{2+}:{\text{S}}_{2}{\text{O}}_{3}{}^{2-}$ mole ratio of 1: 1 and 1: 1.5 are displayed in Figure 8a; while the powder XRD patterns of the powder produced at ${\mathrm{Cu}}^{2+}:{\text{S}}_{2}{\text{O}}_{3}{}^{2-}$ mole ratio of 1: 2.5, 1: 3 and 1: 5 are depicted in Figure 9a.

In general, sharp and narrow peaks are observed from all the diffractograms signifying the formation of highly crystalline compounds. For the synthesis performed at ${\mathrm{Cu}}^{2+}:{\text{S}}_{2}{\text{O}}_{3}{}^{2-}$ mole ratio of 1: 1. However, phase pure natrochalcite [NaCu_2_(SO_4_)(H_2_O)] product with irregular plate-like morphology is detected (Figure 8b). For the synthesis performed at ${\mathrm{Cu}}^{2+}:{\text{S}}_{2}{\text{O}}_{3}{}^{2-}$ mole ratio of 1: 1.5, the diffraction peak of CuS phase is again not detected. Nevertheless, phase pure digenite (Cu_9_S_5_) with irregular platelet-like morphology is in fact identified (Figure 8c). For the synthesis conducted at ${\mathrm{Cu}}^{2+}:{\text{S}}_{2}{\text{O}}_{3}{}^{2-}$ mole ration of 1: 2.5, 1: 3 and 1: 5, both mixed phases of CuS and S_8_ are identified from their powder XRD patterns. In comparison of the products formed at ${\mathrm{Cu}}^{2+}:{\text{S}}_{2}{\text{O}}_{3}{}^{2-}$ mole ration of 1: 2.5, 1: 3 and 1: 5, more intense crystalline S_8_ peaks are detected from the XRD patterns when higher ${\mathrm{Cu}}^{2+}:{\text{S}}_{2}{\text{O}}_{3}{}^{2-}$ mole ratio was applied which indicates that higher amount of S_8_ is present in the system. From the FESEM images (Figure 9b-c), it is amazed to notice that the products obtained at ${\mathrm{Cu}}^{2+}:{\text{S}}_{2}{\text{O}}_{3}{}^{2-}$ mole ration of 1: 2.5 and 1: 3 show crystalline hexagonal plate morphology although certain degree of S_8_ impurity was detected in the system. Nevertheless, the crystalline hexagonal plate morphology is found to be reduced significantly when ${\mathrm{Cu}}^{2+}:{\text{S}}_{2}{\text{O}}_{3}{}^{2-}$ mole ratio was increased to 1: 5 in which a substantial amount of solid mass can be observed from the FESEM image depicted in Figure 9d. From the appealing observations found in this investigation, it can be deduced that the extensive growth of crystalline S_8_ has an inhibiting effect towards the growth of CuS hexagonal plate architecture. The amplified growth of crystalline S_8_ in the synthesis of ${\mathrm{Cu}}^{2+}:{\text{S}}_{2}{\text{O}}_{3}{}^{2-}$ mole ratio of 1: 5 has dominated the development of CuS hexagonal plates. Consequently, this has led to the coverage of crystalline S_8_ on CuS surface which resulted in a more coarsened morphology detected at ${\mathrm{Cu}}^{2+}:{\text{S}}_{2}{\text{O}}_{3}{}^{2-}$ mole ratio of 1: 5.

In this work, efforts have been put to correlate the experimental observations with the chemical reactions occurred. Pryor et. al. reported that ${\text{S}}_{2}{\text{O}}_{3}{}^{2-}$ undergoes irreversible disproportionation in water to produce HS^-^, $\text{S}{\text{O}}_{4}{}^{2-}$and a small amount of S_8_ species as indicated in equation (1) [39].

(1) $$S_{2}O_{3}{}^{2-}+H_{2}O\to {\mathrm{SO}}_{4}{}^{2-}+{\mathrm{HS}}^{-}+H^{+}$$

On the other hand, an examination of the S_8_ species in basic solution revealed that ${\text{S}}_{2}{\text{O}}_{3}{}^{2-}$, HS^-^ and $\text{S}{\text{O}}_{4}{}^{2-}$ species can be produced as shown in the following [40,41]:

(2) $$S_{8}+{8\mathrm{OH}}^{-}↔{2S}_{2}O_{3}{}^{2-}+{4\mathrm{HS}}^{-}+{2H}_{2}O$$

(3) $$S_{8}+10{\mathrm{OH}}^{-}↔{2\mathrm{SO}}_{4}{}^{2-}+{6\mathrm{HS}}^{-}+{2H}_{2}O$$

The reactions shown in equations (2) and (3) are reversible due to the presence of large amount of HS^-^ and $\text{S}{\text{O}}_{4}{}^{2-}$ species in the solution. Thus, the overall reaction which led to the formation of S_8_ species can be summarized as below:

(4) $$S_{2}O_{3}{}^{2-}+{\mathrm{SO}}_{4}{}^{2-}+{5\mathrm{HS}}^{-}+{2H}_{2}O↔S_{8}+{9\mathrm{OH}}^{-}$$

As indicated above, the resulting reaction equilibrium is shifted towards the formation of S_8_ species. Its presence has been evidenced by powder XRD technique at ${\mathrm{Cu}}^{2+}:{\text{S}}_{2}{\text{O}}_{3}{}^{2-}$ mole ratio of 1: 2.5, 1: 3 and 1: 5 during the hydrothermal treatment.

For reaction conducted at ${\mathrm{Cu}}^{2+}:{\text{S}}_{2}{\text{O}}_{3}{}^{2-}$ mole ratio of 1: 1, the formation of NaCu_2_(SO_4_)(H_2_O) with irregular plate-like morphology has been detected. This observation can be explained via the presence of large amount of $\text{S}{\text{O}}_{4}{}^{2-}$ species in the reaction. From equation (4), 5 mole of HS^-^ reacted with 1 mole of $\text{S}{\text{O}}_{4}{}^{2-}$ in the formation of S_8_ species. The consumption of HS^-^ is 5 times higher than $\text{S}{\text{O}}_{4}{}^{2-}$ in the S_8_ species formation pathway. In addition, it is well known that HS^-^ species is reactive and is rapidly oxidized by dissolved oxygen in water to form $\text{S}{\text{O}}_{4}{}^{2-}$ species. Therefore, it is expected that the formation of NaCu_2_(SO_4_)(H_2_O) is more feasible in relative to CuS with the presence of excessive amount of $\text{S}{\text{O}}_{4}{}^{2-}$ species in the system. For synthesis carried out at ${\mathrm{Cu}}^{2+}:{\text{S}}_{2}{\text{O}}_{3}{}^{2-}$ mole ratio of 1: 1.5, digenite (Cu_9_S_5_) with platelet-like morphology was identified. Its formation can be closely related to the slight increased of active HS^-^ present in the solution. An increase in ${\mathrm{Cu}}^{2+}:{\text{S}}_{2}{\text{O}}_{3}{}^{2-}$ mole ratio undeniably elevates the quantity of active HS^-^. It is believed that the increased amount of HS^-^ used to precipitate digenite (Cu_9_S_5_) has outpaced the formation of crystalline S_8_ but it is still insufficient in precipitating CuS. Thus, this has eventually led to the formation of metastable state copper sulphide, namely Cu_9_S_5_ when ${\mathrm{Cu}}^{2+}:{\text{S}}_{2}{\text{O}}_{3}{}^{2-}$ mole ratio of 1: 1.5 was applied in this reaction.

For synthesis conducted at ${\mathrm{Cu}}^{2+}:{\text{S}}_{2}{\text{O}}_{3}{}^{2-}$ mole ratio of 1: 2.5 and 1: 3, mixture of CuS and S_8_ phases with crystalline hexagonal plate morphology has been observed. However, when ${\mathrm{Cu}}^{2+}:{\text{S}}_{2}{\text{O}}_{3}{}^{2-}$ mole ratio was raised to 1: 5, an increase amount of S_8_ phase in relative to CuS phase is detected. From equation (1), it is obvious that an increase amount of ${\text{S}}_{2}{\text{O}}_{3}{}^{2-}$ used will amplify the contribution of $\text{S}{\text{O}}_{4}{}^{2-}$ and HS^-^ species. Subsequently, a greater conversion of ${\text{S}}_{2}{\text{O}}_{3}{}^{2-}$ and $\text{S}{\text{O}}_{4}{}^{2-}$ into S_8_ will be expected based on disproportionation reaction shown in equation (4). The reaction pathway proposed is in good agreement with the product formed because the additional S_8_ phase identified is significantly increased in the powder synthesized at ${\mathrm{Cu}}^{2+}:{\text{S}}_{2}{\text{O}}_{3}{}^{2-}$ mole ratio of 1: 3 and 1: 5. Therefore, it is considerably important to employ an appropriate ${\mathrm{Cu}}^{2+}:{\text{S}}_{2}{\text{O}}_{3}{}^{2-}$ mole ratio for the growth of phase pure CuS hexagonal plates in the reaction.

### Role of reaction time in hydrothermal synthesis

**Figure 10:**
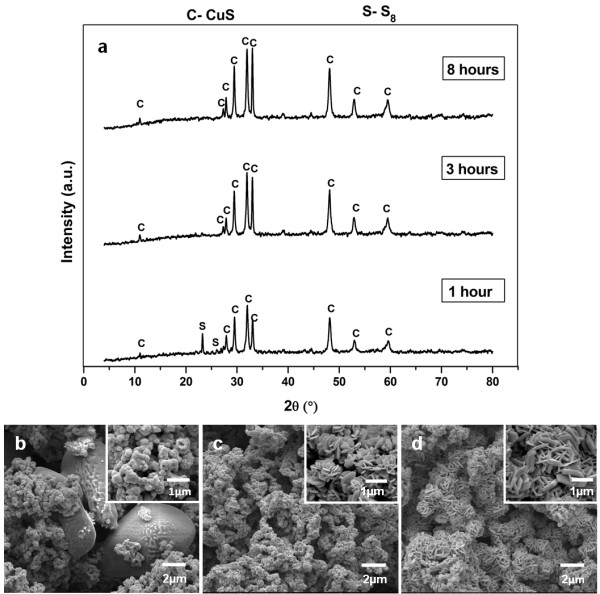
**Products prepared at different reaction time.** Powder XRD patterns (**a**), FESEM images of products synthesized with ${\mathrm{Cu}}^{2+}:{\text{S}}_{2}{\text{O}}_{3}{}^{2-}$ mole ratio of 1: 2 at 155°C for 1 hour (**b**), 3 hours (**c**) and 8 hours (**d**).

For the reaction time studies, investigations were further carried out at synthesis time of 1, 3 and 8 hours to determine the minimum time required to achieve phase pure CuS formation under ${\mathrm{Cu}}^{2+}:{\text{S}}_{2}{\text{O}}_{3}{}^{2-}$ mole ratio of 1: 2 at 155.0°C. The powder XRD patterns and morphology of the products formed at different synthesis time were depicted in Figure 10a-d respectively. At reaction time of 1 hour, phase mixtures of covellite and cyclooctasulphur were found in its powder XRD pattern. This observation is coupled with the absence of hexagonal shaped particles (Figure 10b) as well as the appearance of big solid mass detected from FESEM analysis. This finding strongly suggests that reaction time of 1 hour is insufficient to promote the formation of highly crystalline CuS hexagonal plates and complete decomposition of cyclooctasulphur under the hydrothermal synthesis.

As the reaction time was prolonged to 3 and 8 hours, it can be observed that only covellite phase is remained while cyclooctasulphur has been diminished entirely from the powder XRD patterns. This observation is associated with the single morphology of hexagonal shaped particles identified from FESEM analysis. These results again indicate that the decomposition of cyclooctasulphur occurs during the hydrothermal treatment at 155°C in which reaction time of 3 hours is enough to eliminate this impurity completely. Further analysis of the powder XRD pattern for the pure phase CuS synthesized has been carried out using Scherrer Formula (1918) [42]:

D=kλ/βcosθ

where *D* is the average crystallite size, *k* is the Scherrer constant (0.94), *λ* is the X-ray wavelength (0.15406 nm for CuK_α_), *β* is the angular linewidth of half maximum intensity (FWHM) in radians unit, *θ* is the Bragg angle corresponding to the maximum of the diffraction peak in degrees unit. The average crystallite size of the as-formed CuS phase at reaction time of 3, 8 and typically 12 hours were estimated as (31.49 ± 1.44), (34.80 ± 1.59), and (44.94 ± 2.37) nm respectively. This observation implies that the process of crystallite growth is in favour when synthesis time is lengthened. In fact, the trend of increasing crystallite size with time is also found when comparing the powder XRD patterns of these three products. The diffraction peaks of the respective compounds have become sharper with time, indicating that more crystalline material with larger size was obtained when reaction time was prolonged.

### Formation mechanism of CuS hexagonal plates

From the studies above, it is identified that the formation of CuS crystal phase can be obtained at almost all synthesis conditions studied (variation of ${\mathrm{Cu}}^{2+}:{\text{S}}_{2}{\text{O}}_{3}{}^{2-}$ mole ratio of 1: 2 to 1: 5, reaction temperature of 25 to 155°C and reaction time of 1 to 12 hours). These results indicate that the CuS crystal phase is a thermodynamically stable compound in which under a suitable ${\mathrm{Cu}}^{2+}:{\text{S}}_{2}{\text{O}}_{3}{}^{2-}$ mole ratio, its formation is feasible regardless of the synthesis temperature and reaction time applied. In the reaction between Cu(NO_3_)_2_ and Na_2_S_2_O_3_, it is reasonable to consider that the main CuS formation pathway proceeds firstly via the relatively stable [Cu(S_2_O_3_)(H_2_O)_2_] and [Cu(S_2_O_3_)_2_]^2-^ complexes formation [21,43].

(5) $${\mathrm{Cu}}^{2+}+{2S}_{2}O_{3}{}^{2-}\to {\left[\mathrm{Cu}{\left(S_{2}O_{3}\right)}_{2}\right]}^{2-}$$

(6) $${\mathrm{Cu}}^{2+}+S_{2}O_{3}{}^{2-}+{2H}_{2}O\to \left[\mathrm{Cu}{\left(S_{2}O_{3}\right)}_{2}{\left(H_{2}O\right)}_{2}\right]$$

A subsequent disproportionation reaction takes place in the complexes mentioned above resulting in a complete dissociation of Cu^2+^, SO_4_^2-^ and S^2-^ in the next step.

(7) $$\begin{array}{l} {\left[\mathrm{Cu}{\left(S_{2}O_{3}\right)}_{2}\right]}^{2-}+H_{2}O\to {\mathrm{Cu}}^{2+}+{\mathrm{SO}}_{4}{}^{2-}+S^{2-}\\ \;+{2H}^{+} \end{array}$$

(8) $$\begin{array}{l} \left[\mathrm{Cu}{\left(S_{2}O_{3}\right)}_{2}{\left(H_{2}O\right)}_{2}\right]\to {\mathrm{Cu}}^{2+}+{\mathrm{SO}}_{4}{}^{2-}+S^{2-}\\ \;+{2H}^{+}+H_{2}O \end{array}$$

The key step of CuS formation is suggested to be an in situ reduction of Cu^2+^ to Cu^+^ ion and oxidation of S^2-^ to S^-^ and S_2_^2-^ species when bare S^2-^ is reacting with Cu^2+^ ion.

(9) $$\begin{array}{l} {3\mathrm{Cu}}^{2+}+{3S}^{2-}\to {3\mathrm{Cu}}^{+}+S^{–}+{\left(S_{2}\right)}^{2-}\\ \;\equiv {\left({\mathrm{Cu}}^{+}\right)}_{3}\left(S^{–}\right){\left(S_{2}\right)}^{2-}↓ \end{array}$$

It can be observed that the final reaction shown is not a simple balanced charged reaction that resulted from Cu^2+^ and S^2-^. In fact, the crystal structure of covellite (CuS) has been evidenced by single crystal experiment in which it is composed of (Cu)_3_(S)(S_2_) [44]. The assignment of formal charges of Cu^+^, S^-^ and ${\text{S}}_{2}{}^{2-}$ was actually based on the interpretation from X-Ray photoelectron spectroscopy (XPS) [45]. In addition, the presence of Cu(I) instead of Cu(II) in the crystal lattice was also proven from X-Ray photoelectron (XPS) and X-ray absorption spectroscopic (NEXAFS) studies [33,46]. Taking the monovalency of all copper atoms into account, covellite should not be regarded as Cu (II) sulphide [33]. Therefore, the mechanism deduction which involves the formation of covellite should be based on its molecular formula, (Cu^+^)_3_(S^–^)(S_2_)^2-^ rather than its empirical formula, CuS. The key reaction should consist of a series of redox reactions which build up the core crystal unit of Cu^+^, S^–^ and (S_2_)^2-^ in the final crystal system.

For any crystallite formation, it is renowned that both of the nucleation and growth process play important roles in determining the morphology of the product formed. For the case of CuS hexagonal plate-like architecture, it is believed that its formation is closely related to the crystallographic phase of the seed formed during the nucleation process and its surface selective crystallization in the growth process. The formation of platelet-like CuS seed has been expected in the reaction due to the intrinsic anisotropic characteristics of the CuS hexagonal crystal structure [20,22]. In the subsequent growth stage, the platelet-like seed will grow in a different rate along the planes due to the different surface energy distribution. It was reported that hexagonal metal with a c/a ratio greater than 1.63, surface energy at {101} and {100} surfaces will be 1.5 times greater compared to {001} facets [47]. Similarly, the c/a ratio of CuS is determined to be 4.31 in which an even higher surface energy will be expected at {101} and {100} surfaces [22]. This has induced a relatively fast crystal growth at these facets and resulting in a hexagonal shape particle instead of rod-like particle being observed in the powder [48]. Finally, the formation of stacked-like CuS hexagonal plate morphology can be considered as a typical Ostwald ripening process. Owing to the higher surface energy of small seed crystal, its dissolution and re-deposition onto the larger hexagonal platelet surfaces at different orientation arise during the reaction. The extensive growth of the small seed crystal at bigger dimension of hexagonal plate has resulted in the perpendicular intersection of the platelet-like structure. This process has eventually led to the formation of stacked-like CuS hexagonal plate morphology as depicted in the final powder obtained.

## Conclusions

Via the optimization of reaction parameters in hydrothermal reactions between copper (II) nitrate and sodium thiosulphate without appending any assistant agent, it is found that CuS hexagonal plates can be successfully synthesized at ${\mathrm{Cu}}^{2+}:{\text{S}}_{2}{\text{O}}_{3}{}^{2-}$ mole ratio of 1: 2 under reaction temperature of 155°C for 12 hours. FESEM and TEM examinations confirmed that the CuS hexagonal plates architecture are assembled, stacked and interlaced perpendicular to each other with an mean edge length of 1 μm and thickness of 100 nm. The reactions between copper (II) nitrate and sodium thiosulphate at different synthesis conditions produce a wide range of crystal phases in addition to the covellite (CuS) phase in which a systematic approach is required to decompose the impurities. It is identified that both the reaction temperature and time are important parameters in decomposing the impurities present. Whilst, ${\mathrm{Cu}}^{2+}:{\text{S}}_{2}{\text{O}}_{3}{}^{2-}$ mole ratio is playing a vital role in controlling the amount of cyclooctasulphur (S_8_) present in the final powder obtained. A possible formation mechanism of CuS hexagonal plates based on the presence of Cu (I) instead of Cu (II) species in the crystal structure of covellite (CuS) was also proposed. This facile and mild hydrothermal batch route developed provides a promising new methodology in studying the reaction chemistry of aqueous solution phase reactants as well as synthesizing phase pure CuS hexagonal plates.

## Experimental

### Synthesis and formation studies of CuS hexagonal plates

All reagents used in this study are of analytical grade, obtained from commercial market and were used without further purification. Sodium thiosulphate pentahydrate (Na_2_S_2_O_3_.5H_2_O, Merck) was selected as the sulphur source while copper nitrate pentasemihydrate (Cu(NO_3_)_2_.2½ H_2_O, Riedel de Häen) was used as the copper source. In a typical procedure, CuS was synthesized by reacting 0.025 mol of copper nitrate with 0.05 mol of sodium thiosulphate (${\mathrm{Cu}}^{2+}:{\text{S}}_{2}{\text{O}}_{3}{}^{2-}$ mole ratio = 1: 2.0) in 40.00 ml deionized water. The solutions were mixed thoroughly under constant stirring for 15 minutes; homogeneous slurry was formed, which was then transferred into a sealed 100 ml Teflon-lined stainless steel tube and put into a custom-made rotating furnace. The rotating furnace was maintained at 155.0°C for 12 hours. To measure the synthesis temperature during the reaction, a thermocouple was placed inside the furnace every hour through an injection port without opening the furnace. After 12 hours, the Teflon-lined stainless steel tube was allowed to cool down naturally at ambient temperature. The obtained product was washed with batches of deionized water until the final filtrate showed conductivity of less than 10 μS/cm. The collected product was then dried overnight at ambient temperature in a vacuum desiccator which consists of silica gels as drying agent. After that, the dried sample was purged with purified nitrogen gas for 3 minutes prior storage to prevent the oxidation of the sample [49]. In order to study the role of ${\mathrm{Cu}}^{2+}:{\text{S}}_{2}{\text{O}}_{3}{}^{2-}$ mole ratio, the synthesis was varied in the range of ${\mathrm{Cu}}^{2+}:{\text{S}}_{2}{\text{O}}_{3}{}^{2-}$ mole ratio = 1: 1–1: 5 at 155°C for 12 hours where the number of mole of copper nitrate employed was fixed at 0.025 mol. Meanwhile, the role of synthesis temperature was investigated by controlling the furnace temperature at 25, 65, 95, 125 and 175°C by keeping the other synthesis parameters at constant. The studies on reaction time parameter were examined at 1, 3 and 8 hours with ${\mathrm{Cu}}^{2+}:{\text{S}}_{2}{\text{O}}_{3}{}^{2-}$ mole ratio and synthesis temperature fixed at 1: 2 and 155°C.

### Characterization and analysis of solid samples

The crystalline phase and purity of the synthesized products were identified by powder X-ray Diffraction (XRD), using a Bruker X-ray Diffraction Model D-8 equipped with a Cu Kα monochromatized radiation source (λ = 1.5406 Å) in the range of 4° ≤ 2θ ≤ 80°. Field Emission Scanning Electron Microscopy (FESEM) images and Energy Dispersive X-ray (EDX) were done by using FEI Quanta 200 FESEM. The bulk elemental composition of the material was determined by X-ray Fluorescence (XRF) spectrometer by Bruker (S4-Explorer). TEM and HRTEM images were recorded from a FEI Cs-corrected Titan 80–300 microscope operated at 300 kV. The TG/ DSC results were acquired using NETZSCH STA 449C device coupled with Quadrupole Mass Spectrometer (Thermostar, Balzers). A heating rate of 5°C min^-1^ with mass flow of 30 cm^3^ min^-1^ was applied in investigating the thermal property of the product.

## Competing interests

The authors declare that they have no competing interests.

## Authors’ contributions

YLA has carried out conceptual studies on the formation mechanism of CuS and participated in the manuscript preparation. PLY carried out the synthesis, data analysis on the compounds formed, and helped in the manuscript preparation. XH involved in HRTEM data acquisition and interpretation, as well as manuscript revision. SBAH provided idea and participated in the design of copper sulphide. All authors read and approved the final manuscript.
